# Assessment of disease control rate and safety of sorafenib in targeted therapy for advanced liver cancer

**DOI:** 10.1186/s12957-024-03364-y

**Published:** 2024-04-12

**Authors:** Daolin Zeng, Chunlin Yu, Shiyao Chen, Long Zou, Junjun Chen, Linlong Xu

**Affiliations:** 1 Minimally Invasive Intervention Department, Ganzhou People’s Hospital, Ganzhou, Jiangxi 341000 China; 2 Jiangxi Institute of Applied Science and Technology, Nanchang, Jiangxi 330012 China; 3 Hepatobiliary Surgery Department, Jiangxi Province Cancer Hospital, Nanchang, Jiangxi 330029 China; 4 Key Laboratory of Personalized Diagnosis and Treatment of Nasopharyngeal Carcinoma, National Health Commission (NHC), Jiangxi Cancer Hospital of Nanchang Medical College, Nanchang, Jiangxi 330029 China; 5 Department of Hepato-Biliary-Pancreatic Surgery, Jiujiang First People’s Hospital, 48 Taling South Road Jiujiang, Jiujiang, Jiangxi 332000 China

**Keywords:** Advanced liver cancer, Transarterial chemoembolization, Sorafenib, Disease control rate, Clinical efficacy, Targeted therapy

## Abstract

**Objective:**

The clinical efficacy and safety of sorafenib in patients with advanced liver cancer (ALC) were evaluated based on transarterial chemoembolization (TACE).

**Methods:**

92 patients with ALC admitted to our hospital from May 2020 to August 2022 were randomly rolled into a control (Ctrl) group and an observation (Obs) group, with 46 patients in each. Patients in the Ctrl group received TACE treatment, while those in the Obs group received sorafenib molecular targeted therapy (SMTT) on the basis of the treatment strategy in the Ctrl group (400 mg/dose, twice daily, followed by a 4-week follow-up observation). Clinical efficacy, disease control rate (DCR), survival time (ST), immune indicators (CD3+, CD4+, CD4+/CD8+), and adverse reactions (ARs) (including mild fatigue, liver pain, hand-foot syndrome (HFS), diarrhea, and fever) were compared for patients in different groups after different treatments.

**Results:**

the DCR in the Obs group (90%) was greatly higher to that in the Ctrl group (78%), showing an obvious difference (*P* < 0.05). The median ST in the Obs group was obviously longer and the median disease progression time (DPT) was shorter, exhibiting great differences with those in the Ctrl group (*P* < 0.05). Moreover, no great difference was observed in laboratory indicators between patients in various groups (*P* > 0.05). After treatment, the Obs group exhibited better levels in all indicators. Furthermore, the incidence of ARs in the Obs group was lower and exhibited a sharp difference with that in the Ctrl group (*P* < 0.05).

**Conclusion:**

SMTT had demonstrated good efficacy in patients with ALC, improving the DCR, enhancing the immune response of the body, and reducing the incidence of ARs, thereby promoting the disease outcome. Therefore, it was a treatment method worthy of promotion and application.

## Introduction

Liver cancer (LC) is a malignant tumor that occurs in liver cells. It is commonly classified into two types: primary LC and metastatic LC [1, 2]. The etiology of LC is diverse, but the most common risk factors include chronic viral hepatitis (such as hepatitis B and C virus infection), cirrhosis, long-term alcohol consumption, obesity, and diabetes [3, 4]. Genetic factors, environmental exposure, and dietary habits may also play a role in the development of LC. Early-stage LC may not present obvious symptoms and is often detected at advanced stages [5, 6]. Common symptoms include abdominal mass or swelling, upper abdominal pain, jaundice, decreased appetite, weight loss, and fatigue. If these symptoms occur, it is advisable to seek medical attention promptly for further examination and diagnosis [7]. The treatment approach for LC depends on the severity of the condition and the overall health of the patient. Common treatment options include surgical resection, liver transplantation, radiation therapy, chemotherapy, and targeted therapy [8]. The goals of treatment are typically to control tumor growth, alleviate symptoms, prolong survival time, and improve the quality of life (QOL) of patients.

Currently, LC mainly can be treated by surgical treatment, liver transplantation, ablation therapy, interventional therapy, radiation therapy, chemotherapy, and targeted therapy [9]. Among them, transarterial chemoembolization (TACE) is a surgical procedure that involves catheterization of the femoral artery, guiding the catheter through the abdominal aorta to the hepatic arteries, and delivering chemotherapy drugs directly into the tumor lesions. It is characterized by minimal invasiveness, high safety, and fast recovery. TACE is one of the commonly used non-surgical treatment options for primary LC, including hepatic arterial infusion chemotherapy and transcatheter arterial embolization [10]. The procedure involves catheterization of the femoral artery, advancing it to the liver lobe or segmental artery to administer chemotherapy drugs, followed by the injection of embolic agents such as emulsified iodized oil and gelatin sponge into the tumor artery to block the blood supply, leading to tumor ischemic necrosis [11]. TACE has rapidly developed and become the preferred treatment for unresectable LC. It is contraindicated only in cases of complete portal vein occlusion and severe cirrhosis with high portal vein pressure, while the majority of patients can undergo TACE treatment [12].

The pharmaceutical treatment methods for LC primarily include chemotherapy, targeted therapy, and immunotherapy. Chemotherapy involves the use of anticancer drugs to kill or inhibit the growth of cancer cells [13, 14]. In the treatment of LC, commonly used chemotherapy drugs include sorafenib, regorafenib, and cabozantinib [15, 16]. These drugs can suppress the growth of LC by inhibiting tumor angiogenesis, suppressing cell proliferation, and inducing cell apoptosis. Targeted therapy refers to the use of specific drugs to interfere with specific biomolecules within cancer cells, thereby inhibiting tumor growth and spread. Sorafenib is the most commonly applied targeted therapy drug for hepatocellular carcinoma patients [17]. Additionally, ramucirumab and lenvatinib are also targeted drugs used in the treatment of LC. Immunotherapy aims to activate the patient’s own immune system to fight against cancer cells. In the treatment of LC, anti-PD-1 antibody drugs such as pembrolizumab and nivolumab are used to treat advanced LC patients [18, 19]. These drugs can help restore the ability of immune system to attack tumors. In addition to monotherapy, combination therapy may also be used at times to enhance treatment efficacy. The selection of the pharmaceutical treatment regimen is personalized and based on factors such as the stage of LC, the patient’s overall health, and other considerations [20].

Sorafenib (trade name: Nexavar) belongs to the class of molecular targeted therapy drugs and exerts a dual action of inhibiting angiogenesis and tumor cell proliferation. In the past two years, sorafenib treatment for LC has received significant attention, and breakthrough progress has been made in clinical research. The ability to effectively prevent disease progression and greatly prolong the survival time (ST) of LC patients. Sorafenib has emerged as a novel and effective drug and treatment method, ushering in a new era of targeted therapy for LC [21]. Clinical studies have found that sorafenib inhibits the growth of LC by suppressing tumor angiogenesis and cell proliferation. It acts on multiple signaling pathways, including vascular endothelial growth factor receptors (VEGFR) and fibroblast growth factor receptors (FGFR) on tumor cells, as well as platelet-derived growth factor receptors (PDGFR) and Raf kinases. Sorafenib is typically administered orally, and the dosage and treatment schedule are adjusted according to the doctor’s recommendations [22]. Possible side effects may include fatigue, loss of appetite, diarrhea, hand-foot syndrome (HFS) characterized by redness, swelling, and pain, and hypertension. These side effects may vary depending on individual differences, so close collaboration with the doctor is crucial [23, 24]. Currently, sorafenib has been extensively utilized in treating LC and has demonstrated the benefit of prolonging the ST of patients. However, individual patient responses and tolerability may vary, so the treatment effect and duration will differ based on individual differences. During the treatment process, doctors regularly monitor disease progression and side effects and make adjustments and management decisions accordingly [25].

In conclusion, there are various clinical diagnostic and treatment methods available for advanced LC (ALC), and the selection of targeted therapy drugs is crucial. Based on this, this work selected 100 ALC patients admitted to the hospital from May 2020 to August 2022 as research subjects. They were rolled into two groups using a random number table method: an observation group (Obs group) of 50 patients and a control group (Ctrl group) of 50 patients. In the Ctrl group, patients received TACE alone, while in the Obs group, patients received oral sorafenib in addition to TACE. By comparing the basic information, disease control rate (DCR), QOL, immune indicators, adverse reactions (ARs), and other factors between the two groups, the clinical efficacy and safety of adding sorafenib to TACE in ALC patients were investigated in depth.

## Materials and methods

### Research objects

100 patients diagnosed with ALC and admitted to Jiujiang First People’s Hospital from May 2020 to August 2022 were selected and grouped into an Obs group and a Ctrl group, with 50 patients in each. The random allocation was performed using a random number table.

Prior to their inclusion in the study, the selected patients were provided with detailed information about the research objectives, procedures, potential risks, and benefits. They were also informed that their participation was voluntary and that they could withdraw from the study at any time without affecting their regular medical care. If the patients agreed to participate and their family members consented, they were asked to sign an informed consent form. The implementation of this study has received approval from the Hospital Ethics Committee to ensure that ethical considerations and patient rights are protected throughout the research process.

According to the following criteria, the patients were enrolled: (I) patients diagnosed with LC based on pathological tissue examination; (II) patients with available complete clinical data; (III) patients with primary LC; (IV) patients who had not received any relevant treatment for LC; (V) patients who were not pregnant women; and (VI) patients who were diagnosed as stage B or stage C according to the Barcelona Clinic Liver Cancer (BCLC) staging system.

The patients had to be excluded if they had any of following conditions: (I) patients with concurrent mental abnormalities; (II) patients who voluntarily refused treatment; (III) patients with renal dysfunction; and (IV) patients with allergies to fluorouracil glucose injection, sorafenib, or other specified medications.

### BCLC staging system

The introduction of BCLC staging system will help assess the disease status of patients, provide accurate treatment plans, and predict patient prognosis. The assessment criteria of the system are outlined in Table 1, where a score of 0 indicated fully normal functional capacity, with no difference compared to pre-onset activity level. A score of 1 indicated the ability to walk and engage in light physical activities, including regular household chores or office work, but not heavy physical activities. A score of 2 indicated the ability to walk and perform self-care but a loss of work capacity, with the ability to get out of bed and move for at least half of the daytime. A score of 3 indicated partial self-care, spending more than half of the daytime in bed or in a wheelchair. A score of 4 indicated being bedridden and unable to perform self-care activities.

**Table 1:** The assessment criteria of the BCLC staging system

Stage	Tumor condition	Score
A1	1 tumor	0
A2	1 tumor	0
A3	1 tumor	0
A4	3 tumors with a diameter smaller than 3 cm.	0
B	Multiple tumors	0
C	Multiple tumors with vascular invasion or extrahepatic metastasis	1–2
D	Any tumor stage	3–4

### TACE method

The percutaneous puncture technique was used to assess the size, location, number, and blood supply of the lesions in all patients. Subsequently, the patients were administered 150 mg of oxaliplatin, 40 mg of bevacizumab, and 1 g of 5-fluorouracil (5-FU). The selection of the embolization method and the dosage of iodized oil for arterial embolization is based on the size of the lesions, with injection doses typically ranging from 6 to 25 mL.

### Sorafenib administration

The patients in the Obs group, totaling 50 individuals, were administered oral sorafenib. The initial dosage of the medication was 400 mg twice daily. In the event of ARs, the dosage should be reduced according to the National Cancer Institute’s Common Terminology Criteria for Adverse Events (NCI-CTC). The reduced dosage would be once daily, 400 mg per dose. Subsequently, it would be administered every other day, still at a dosage of 400 mg. Once the ARs subside, the regular dosage can be resumed.

The NCI-CTC was provided in Table 2, which included a grading scale ranging from 0 to 4.

**Table 2:** NCI-CTC

Item	Grade	Explanations
Nausea	0	No nausea events occurred.
	1	Some experienced mild nausea but it did not affect their appetite, and their food intake remained normal.
	2	Some experienced mild nausea but were still able to eat, although their food intake decreased.
	3	Severe nausea was present, making it impossible to eat.
Peripheral neurotoxicity	0	Normal reflexes observed.
	1	Diminished tendon reflexes.
	2	Severe sensory abnormalities and mild weakness.
	3	Significant motor impairment noted.
	4	Patient is paralyzed.
Oral ulceration	0	Patient shows no abnormal signs.
	1	Mild ulceration or erythema, but no pain reported.
	2	Erythema with swelling and pain, ulceration present but still able to eat.
	3	Erythema with swelling and pain, extensive ulceration, unable to eat.
	4	Erythema with swelling and pain, extensive ulceration, unable to eat, requiring urgent nutritional support treatment.
Vomiting	0	No vomiting reported.
	1	Vomiting once within a day.
	2	Vomiting 2–5 times within a day.
	3	Vomiting 6–10 times within a day.
	4	Vomiting more than 10 times within a day, requiring intravenous fluid therapy.

### Observation indicators

(1) General information from both groups of patients will be collected, including age, gender, disease duration, BCLC staging, tumor diameter, tumor thrombus, and extrahepatic metastasis.

(2) The clinical efficacy of both groups was evaluated based on disease progression (tumor volume increase by more than 20% or the occurrence of distant metastasis), disease stabilization (tumor volume reduction of no more than 29% or tumor volume increase of no more than 20%), partial remission (tumor volume reduction of 30% or more), and complete remission (disappearance of the tumor). The DCR was calculated and analyzed.

(3) The Karnofsky Performance Score was based to assess the QOL of patients in different groups. An increase in score greater than 10 points indicated a significant improvement in QOL, an increase or decrease within 10 points indicated a stable quality of life, and a decrease greater than 10 points suggested a decline in QOL.

(4) Fasting venous blood samples was collected from patients. Immunological markers, including CD3+, CD4+, and CD4+/CD8 + lymphocyte subpopulations, were measured using flow cytometry before and after the patients were treated.

(5) Follow-up records will be kept for ARs experienced by patients in both groups. These events may include mild fatigue, liver pain, HFS, diarrhea, and fever. The incidence of ARs was calculated eventually.

### Methods for statistics

The data were analyzed using SPSS 20.0. Descriptive statistics, such as mean ± standard deviation (‾x ± s), would be used to represent continuous variables, while percentages (%) were applied for categorical variables. Group comparisons was performed using repeated measures analysis of variance for between-group comparisons and two-way ANOVA for within-group comparisons. A two-tailed test with *P* < 0.05 was considered statistically significant.

## Results

### Clinical outcomes of patients in different groups

According to Table 3, no remarkable differences were observed between the Obs group and Ctrl group in terms of age, gender, disease duration, BCLC staging, tumor diameter, cancer embolus, and extrahepatic metastasis (*P* > 0.05).

**Table 3:** Clinical outcomes of patients in different groups

Indicator			Obs group (*n* = 50)	Ctrl group (*n* = 50)	P
Gender		Males	27	29	> 0.05
		Females	23	21	
Age (years old)			56.13 ± 4.72	54.58 ± 5.37	> 0.05
Disease duration (years)			1.89 ± 0.45	2.04 ± 0.39	> 0.05
BCLC staging	B		20	18	> 0.05
	C		30	32	
Tumor dimeter (cm)	>= 5		38	39	> 0.05
	< 5		12	11	
Cancer embolus	Yes		29	28	> 0.05
	No		21	22	
Extrahepatic metastasis	Yes		33	31	> 0.05
	No		7	9	

### DCR of patients after different treatments

**Fig. 1:**
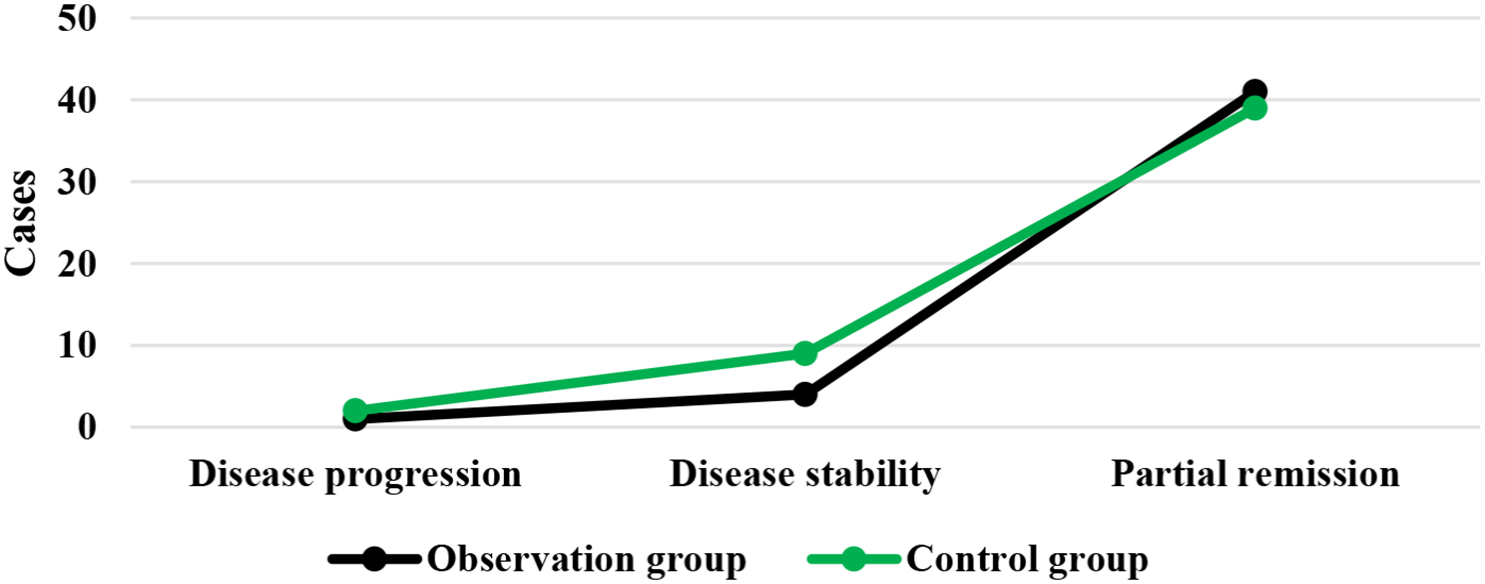
Numbers of patients with disease progression, stability, and partial remission in different groups

**Fig. 2:**
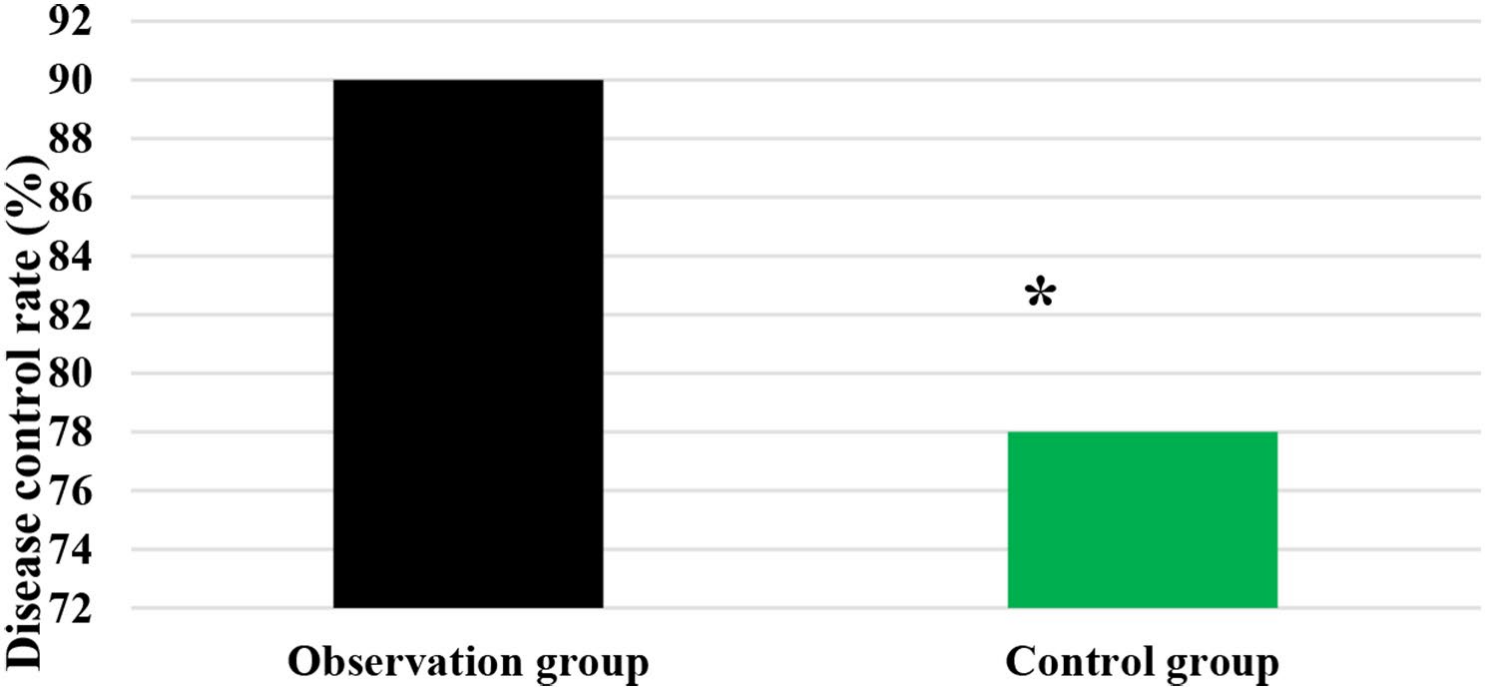
Comparison on DCR of patients after treatment. *Note* * suggested a great difference with *P* < 0.05 to the Ctrl group

As demonstrated in Figs. 1 and 2, in the Obs group, there was disease progression in 1 case, disease stability in 4 cases, and partial remission in 41 cases, showing a DCR of 90%. In the Ctrl group, there was disease progression in 2 cases, disease stability in 9 cases, and partial remission in 39 cases, with a DCR of 78%. It is evident that the Obs group had a sharp higher DCR and exhibited a great difference with the Ctrl group (*P* < 0.05).

### Median ST and median DPT of patients

**Fig. 3:**
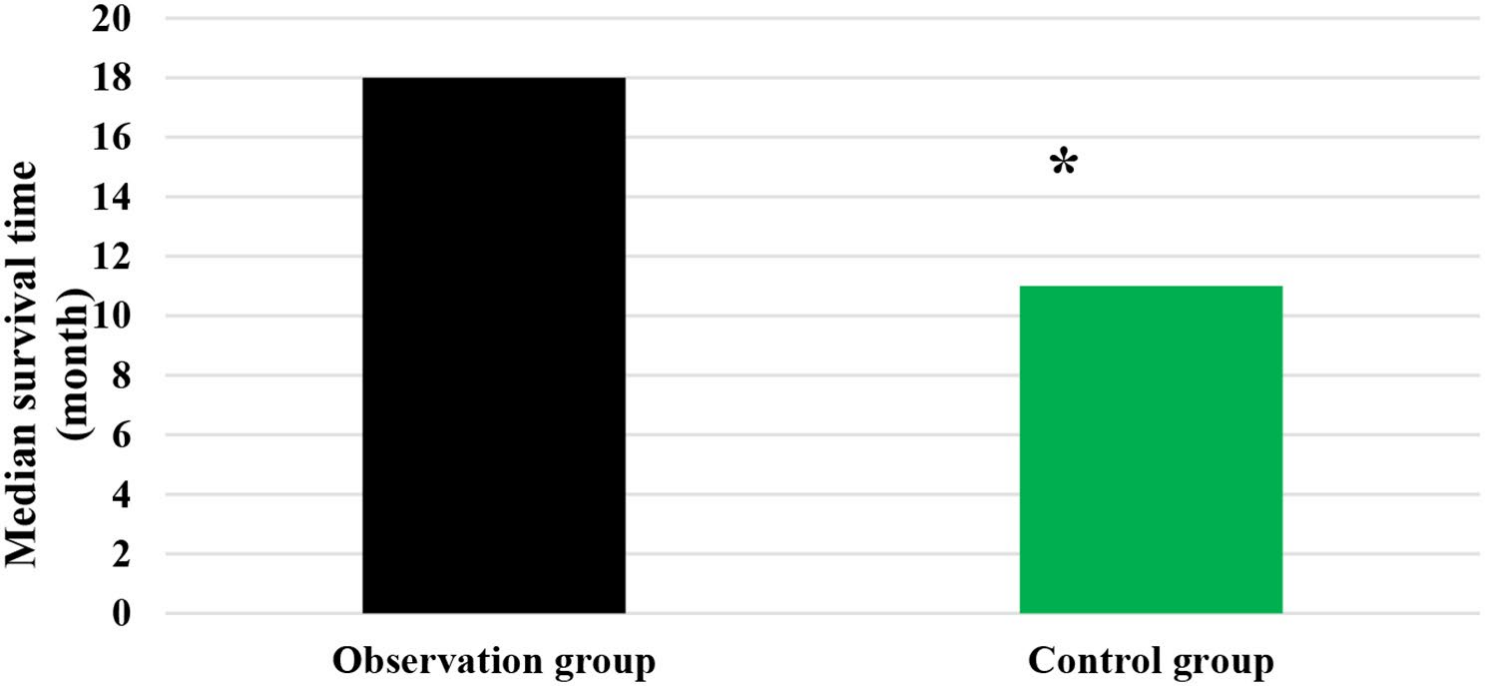
Comparison of median ST of patients in different groups. *Note* * suggested a great difference with *P* < 0.05 to the Ctrl group

**Fig. 4:**
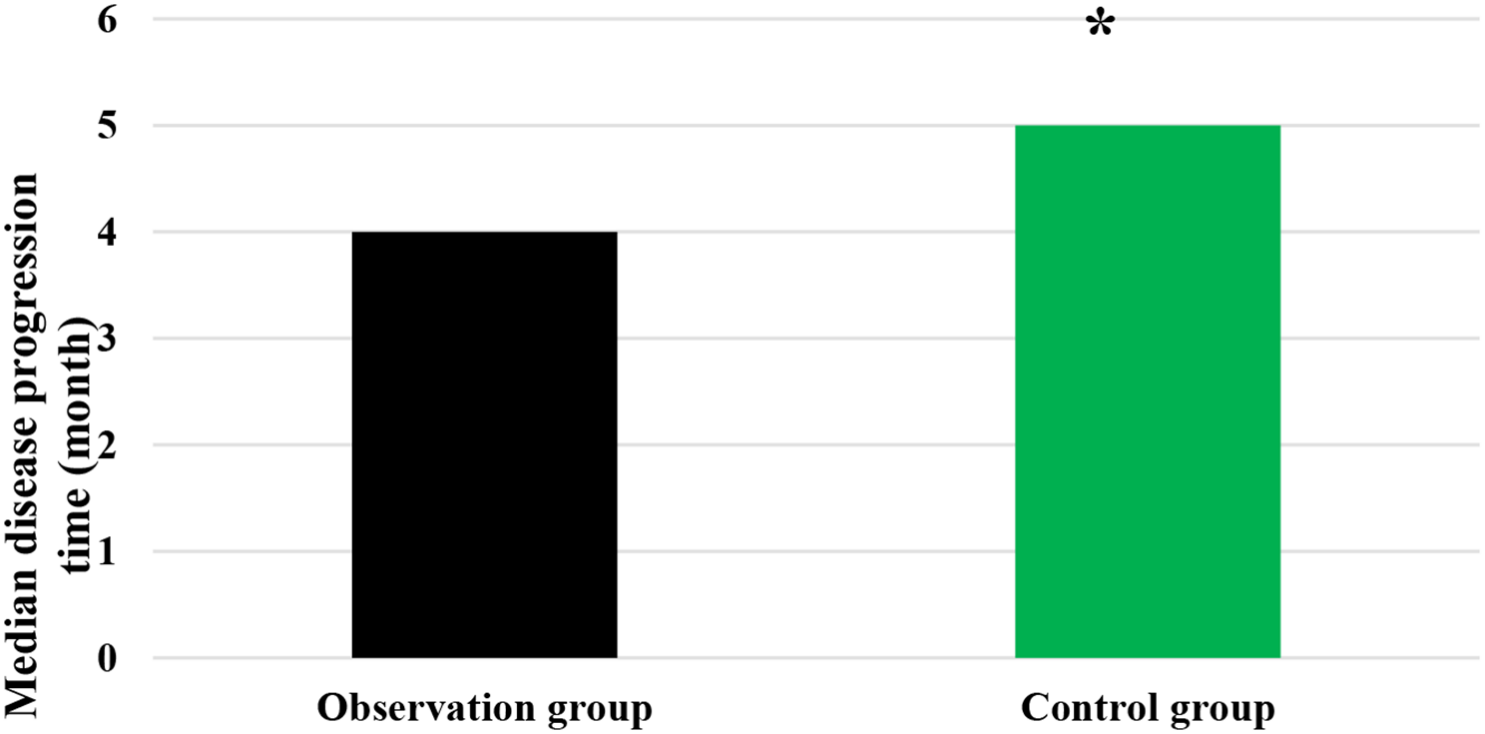
Comparison of median DPT of patients in different groups. *Note* * suggested a great difference with *P* < 0.05 to the Ctrl group

As compared in Figs. 3 and 4, the median ST and median DPT in the Obs group were 18 months and 4 months, respectively; while those in the Ctrl group were 11 months and 5 months, respectively. It is evident that the Obs group exhibited a higher median ST and a lower median DPT than the Ctrl group, showing statistically obvious differences (*P* < 0.05).

### Comparison on QOL of patients after different treatments

**Fig. 5:**
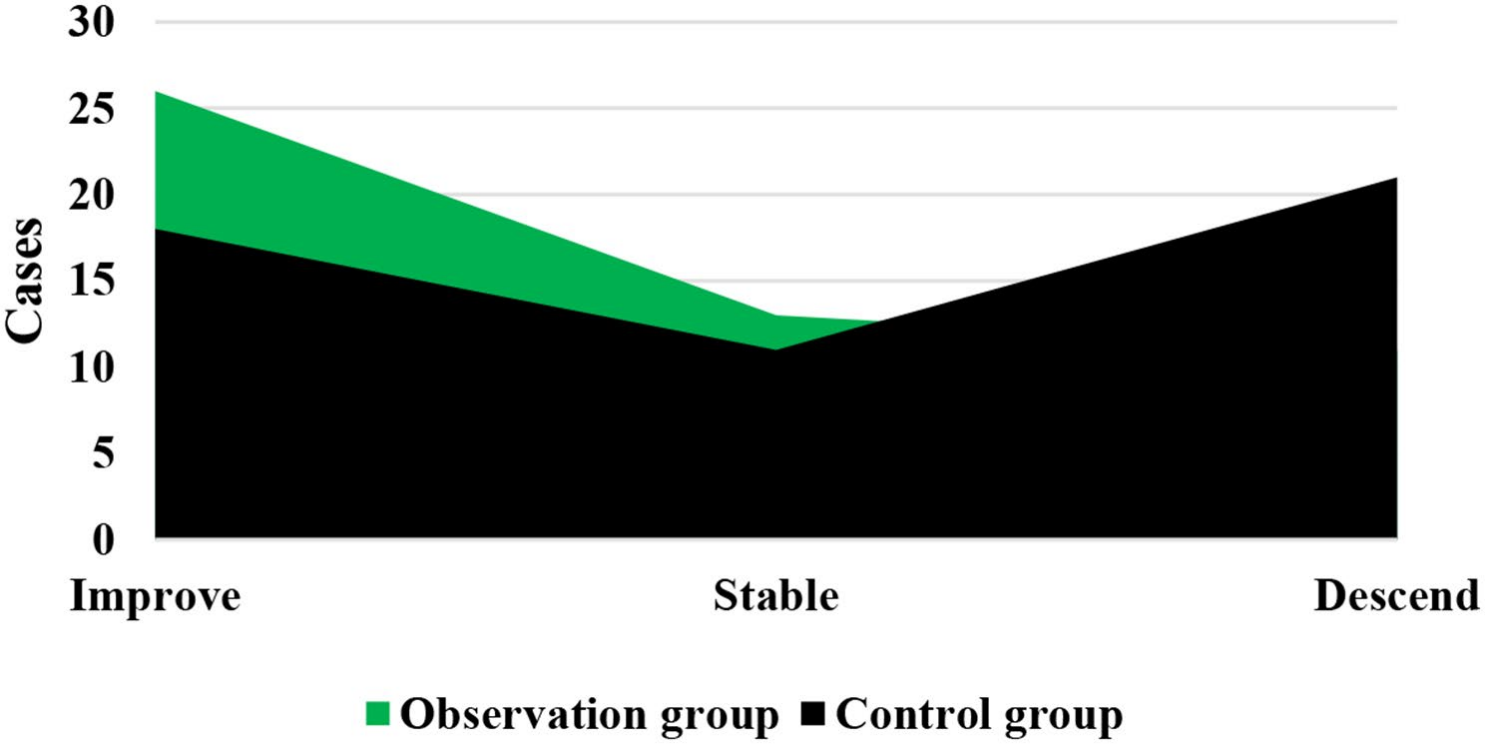
Comparison on number of patients with improved, stable, and decreased QOL after treatment

**Fig. 6:**
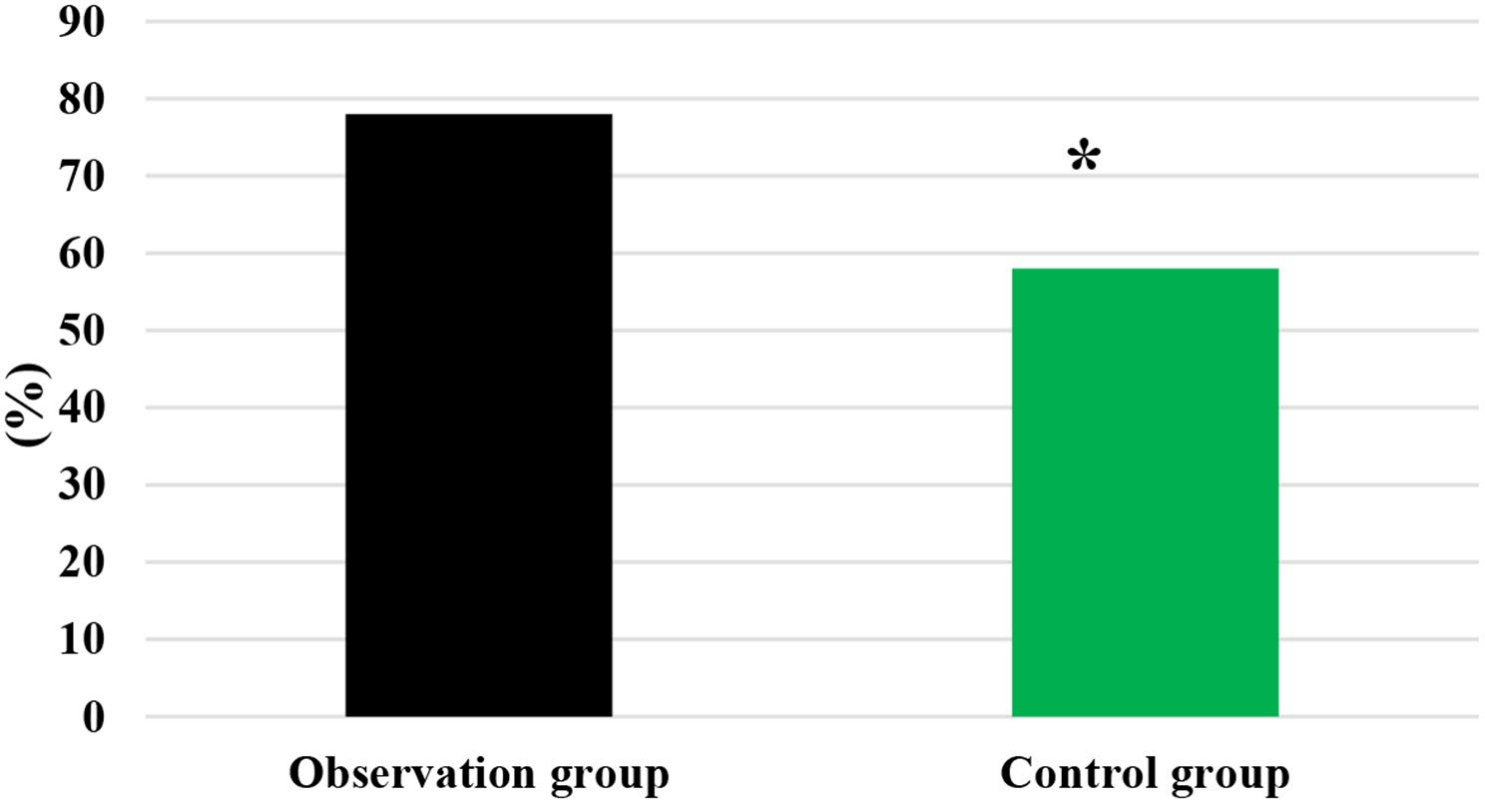
Comparison on rate of improved and stable QOL of patients in various groups. *Note* * suggested a great difference with *P* < 0.05 to the Ctrl group

The QOL of patients was compared after they received different treatment methods, as illustrated in Figs. 5 and 6. In the Obs group, there were 26, 13, and 11 cases with improved, stable, and decreased QOL, respectively. In the Ctrl group, there were 18, 11, and 21 cases with improved, stable, and decreased QOL, respectively. The Obs group had a much higher rate of improved and stable QOL compared to the Ctrl group ((78% vs. 58%), showing a marked difference with *P* < 0.05.

### Changes in immune indicators of patients before and after they were treated

**Fig. 7:**
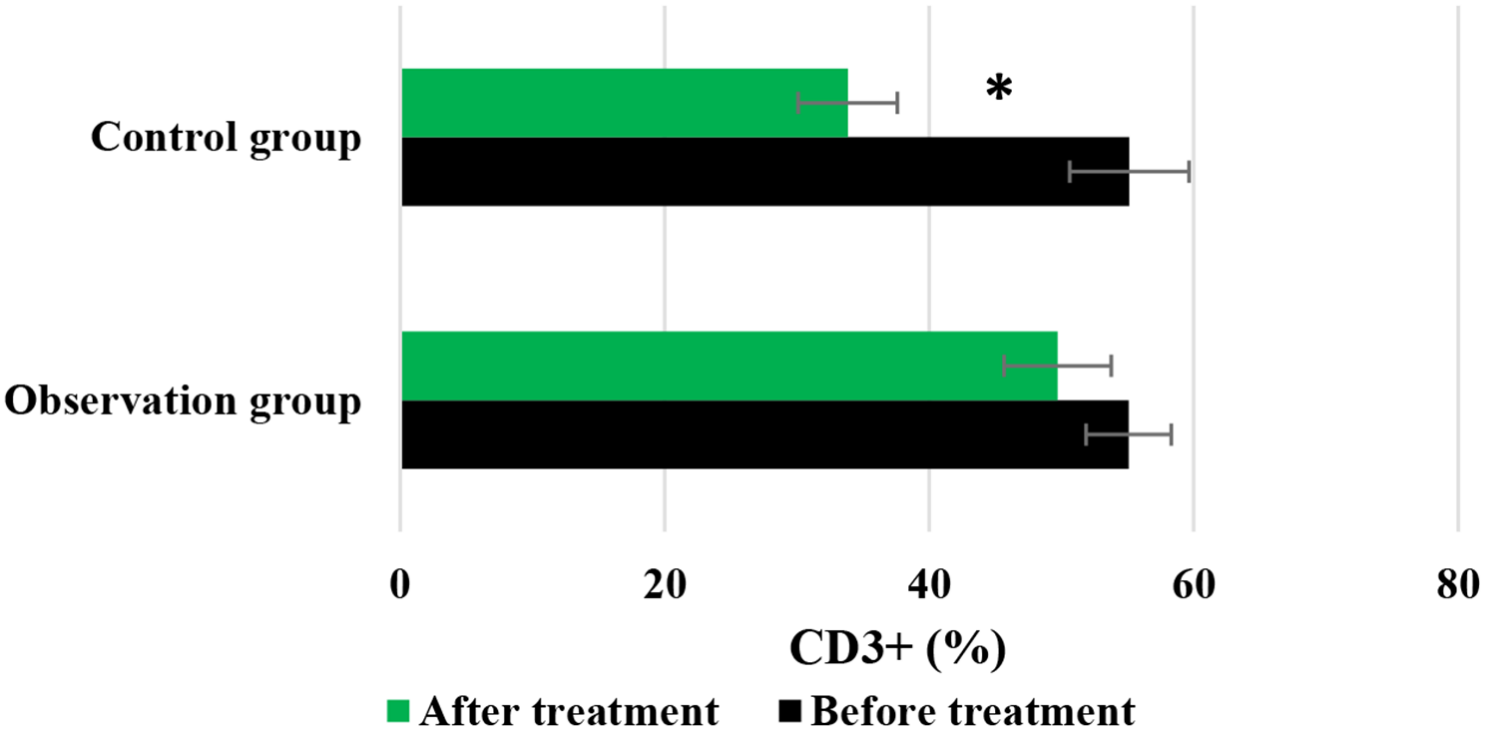
Changes in CD3 + of patients before and after different treatments. *Note* * suggested a great difference with *P* < 0.05 to the Ctrl group

**Fig. 8:**
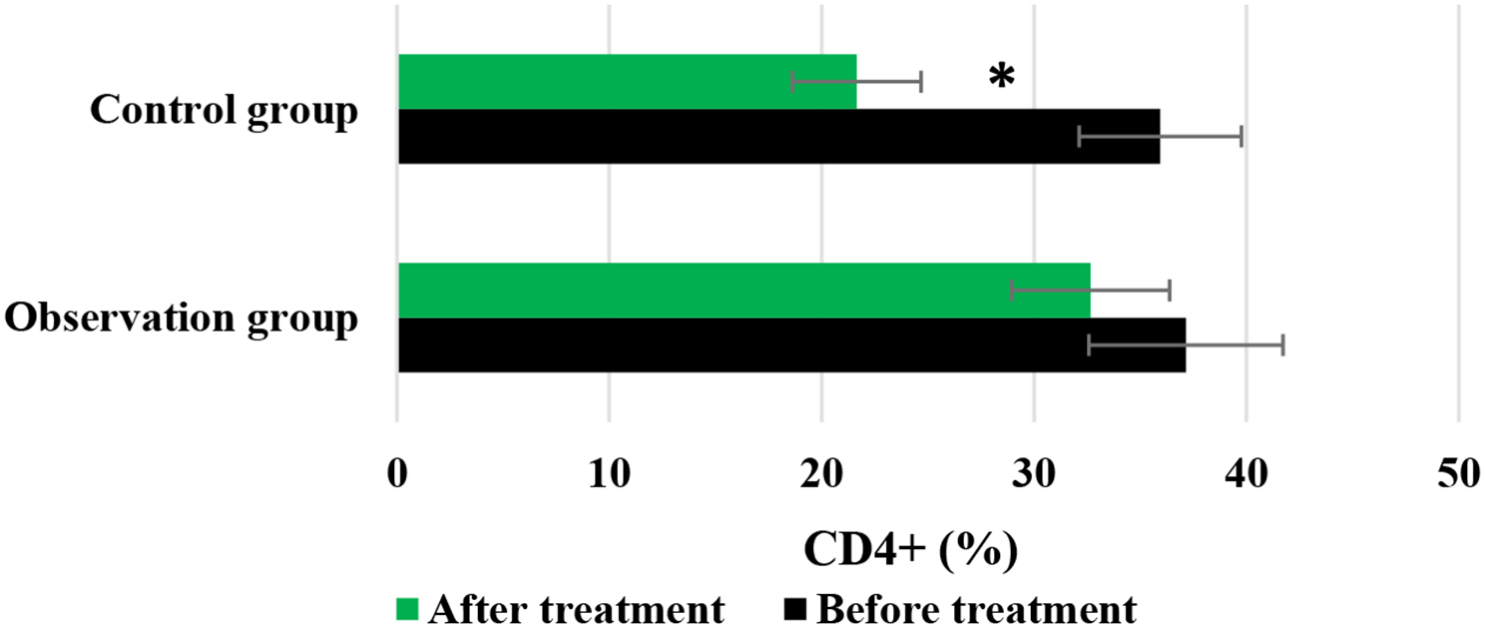
Changes in CD4 + of patients before and after different treatments. *Note* * suggested a great difference with *P* < 0.05 to the Ctrl group

**Fig. 9:**
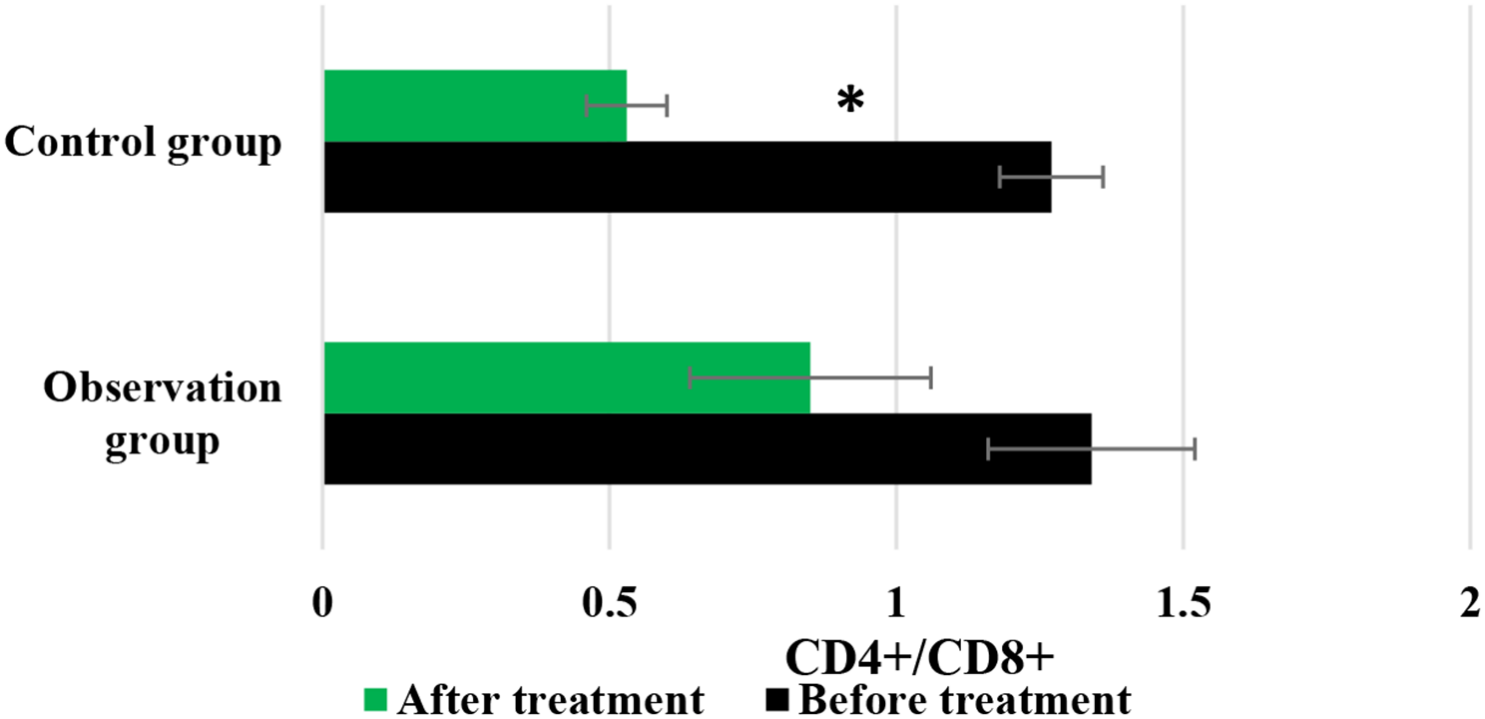
Changes in CD4+/CD8 + of patients before and after different treatments. *Note* * suggested a great difference with *P* < 0.05 to the Ctrl group

Figures 7 and 8, and 9 compared the changes in CD3+, CD4+, and CD4+/CD8 + of patients before and after they were treated differently, respectively. In the Obs group, the levels of CD3+, CD4+, and CD4+/CD8 + before treatment were 55.08 ± 3.22%, 37.15 ± 4.57%, and 1.34 ± 0.18%, respectively; while the posttreatment levels of above indicators were 49.71 ± 4.05%, 32.66 ± 3.72%, and 0.85 ± 0.21%, respectively. In contrast, for patients in the Ctrl group, the pretreatment levels of above three indicators were 55.13 ± 4.51%, 35.94 ± 3.82%, and 1.27 ± 0.09%, respectively; while the level after treatment were 33.84 ± 3.75%, 21.65 ± 3.03%, and 0.53 ± 0.07%, respectively. No visible differences were observed in the levels of CD3+, CD4+, and CD4+/CD8 + between the Obs group and Ctrl group before the patients were treated (*P* > 0.05). However, the posttreatment levels of these indicators in the Obs group were greatly higher to those in the Ctrl group, showing obvious differences with *P* < 0.05.

### Comparison on incidence of ARs of patients in different groups

**Fig. 10:**
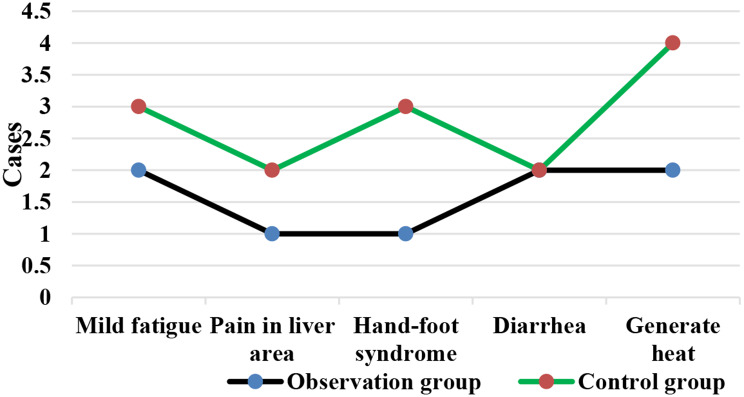
Number of patients with posttreatment mild fatigue, liver pain, HFS, diarrhea, and fever

**Fig. 11:**
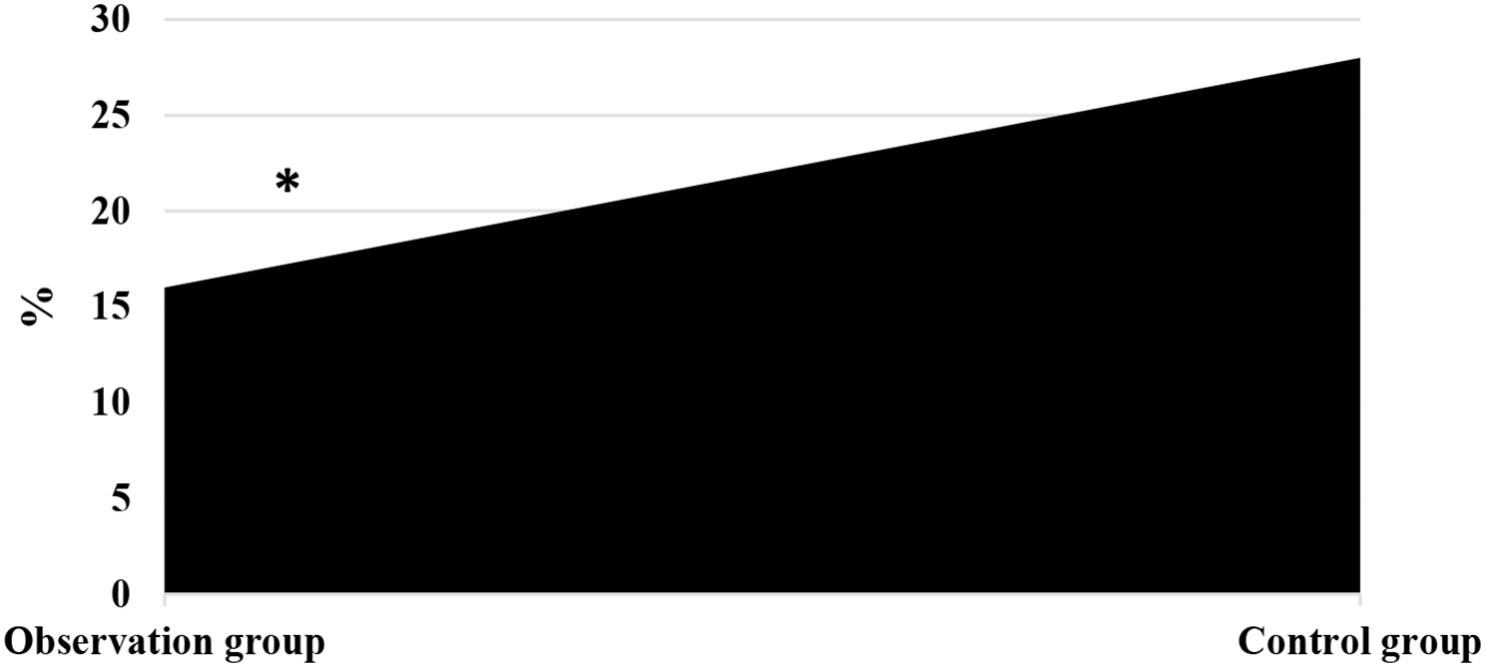
Comparison on posttreatment incidence of ARs in different groups. *Note* * suggested a great difference with *P* < 0.05 to the Ctrl group

As illustrated in Fig. 10, in the Obs group, there were 2 cases of mild fatigue, 1 case of liver pain, 1 case of HFS, 2 cases of diarrhea, and 2 cases of fever. In the Ctrl group, there were 3 cases of mild fatigue, 2 cases of liver pain, 3 cases of HFS, 2 cases of diarrhea, and 4 cases of fever. The Fig. 11 revealed that the incidence of ARs in the Obs group (16%) was much lower to that in the Ctrl group (28%), showing a great difference (*P* < 0.05).

## Discussion

Cancer poses a threat to the health of many individuals. Among various types of cancer, LC is considered the most dangerous because it often presents no symptoms, and patients are typically diagnosed at an advanced stage. Additionally, in China, LC accounts for half of the global incidence, with over 70% of patients being diagnosed in the middle to late stages [26]. This phenomenon makes LC even more frightening. LC refers to malignant tumors that originate in or spread to the liver. Symptoms of LC include a lump or pain in the lower right side of the ribcage, ascites, jaundice, easy bruising, weight loss, and overall weakness [27]. The primary causes of LC are hepatitis B, hepatitis C, and alcohol-induced cirrhosis. Other causes include aflatoxin, non-alcoholic fatty liver disease, and liver fluke infection. In China, LC is prevalent, and most patients are diagnosed in the middle to late stages, losing the opportunity for surgical intervention. With advancements in medical technology, the available treatment options for LC have been rapidly evolving [28, 29]. Due to its insidious onset, atypical early symptoms, and diagnostic challenges, most patients with LC are already in the late stage or have distant metastasis at the time of diagnosis, with only about 15% of patients being suitable for surgical resection. For late-stage patients who have lost the opportunity for surgery, drug therapy becomes the main treatment approach. Even for those who can undergo surgical resection, postoperative recurrence is common, requiring adjuvant drug therapy [30]. However, treating LC is particularly challenging due to the presence of hepatitis, cirrhosis, and liver dysfunction in patients. Interventional treatments are effective but invasive and often limited in their application. Currently, there is no evidence to suggest that chemotherapy can prolong survival in LC, and there is a lack of universally recognized effective drugs and standardized regimens. As a result, late-stage patients often face the dilemma of having no effective treatment options and a poor prognosis. Currently, the main treatment methods for LC include surgery, liver transplantation, ablation therapy, interventional therapy, radiation therapy, chemotherapy, targeted therapy, and immunotherapy. Among these, targeted therapy acts like a missile, precisely attacking the tumor while minimizing harm to normal cells. Therefore, compared to chemotherapy, targeted therapy generally offers better control of ARs [31]. In this work, 100 patients with ALC admitted to the hospital from May 2020 to August 2022 were rolled into two groups using a random number table: 50 patients in the Obs group and 50 patients in the Ctrl group. The patients in the Ctrl group received TACE alone, while those in the Obs group received both TACE and oral sorafenib treatment. The balance of baseline data between the groups was ensured to guarantee comparability of the observed results of the dependent variables under similar baseline conditions, thereby examining the true impact of the intervention factors on the observed results. A comparison of the baseline characteristics between the two groups revealed no statistically great differences in age, gender, disease duration, BCLC stage, tumor diameter, cancer thrombus, or extrahepatic metastasis (*P* > 0.05). This provides feasibility for the subsequent comparison of data between the groups.

DCR refers to the proportion of patients whose tumors shrink or remain stable for a certain period of time, including complete remission, partial remission, and stabilization cases [32]. It was found that the DCR of patients in the Obs group (90%) was sharply higher than that in the Ctrl group (78%), showing a difference with *P* < 0.05. This indicates that the addition of sorafenib treatment on top of TACE has a significant short-term efficacy for patients with ALC, effectively inhibiting the occurrence and progression of tumors. The median STof patients in the Obs group was higher and the median DPT was lower, exhibiting substantial differences to those in the Ctrl group (*P* < 0.05). The median ST represents the time at which only 50% of individuals can survive beyond that duration. When evaluating the median survival time for a specific cancer type, it is generally calculated from the time of tumor detection. DPT refers to the time from randomization to objective tumor progression [33, 34]. Therefore, these results indicate that the addition of sorafenib treatment on top of TACE can effectively prolong the ST of patients with ALC and delay the progression of the disease.

The study of QOL in medical research has received high attention and has reached a high level of investigation, with widespread applications. The use of QOL measurements allows for the evaluation of the health status of populations and the QOL of patients with various diseases. It also helps assess the effectiveness of optimizing clinical treatment interventions and various preventive healthcare measures, as well as exploring factors influencing health and disease prevention priorities and participating in decision-making regarding the allocation and utilization of healthcare resources [35, 36]. In this work, the rate of improvement and stability in QOL for patients in the Obs group (78%) was higher, showing a remarkable difference to that in the Ctrl group (58%) (*P* < 0.05). This indicates that the addition of sorafenib treatment on top of TACE can effectively improve the QOL for patients with ALC. Immunity, also known as resistance, refers to the coordinated functioning of various systems in the human body under the control of the central nervous system to ensure the normal functioning of life activities [37]. The immune system is a crucial component of this coordination, with tissue barriers acting as the first line of defense of the immune system. Within the immune cells, CD4 + factors can secrete cellular tissue to enhance immune response and kill tumor cells, while CD8 + factors can secrete inhibitory cells to suppress CD4 + factors, inhibit B cell synthesis, and thereby inhibit immune response, resulting in decreased immune function [38]. This work compared immune function indicators between patients in different groups and found that the levels of CD3+, CD4+, and CD4+/CD8 + in patients in the Obs group after treatment were much higher, with *P* < 0.05 to the levels of these indicators in the Ctrl group. This suggests that the addition of sorafenib treatment on top of TACE can greatly enhance the immune capacity of patients with LC and improve their immune function [39]. Safety is a prerequisite for clinical drug use, and the basic principle is that drugs should not cause or only cause minor and acceptable ARs or side effects. For example, family planning drugs and drugs for infants and young children have high safety requirements, while drugs for life-threatening tumors and AIDS may allow for certain ARs [40]. As a multi-targeted molecular targeted drug, sorafenib, like other drugs, may have certain toxic and side effects, with gastrointestinal bleeding, rash, hypertension, hair loss, diarrhea, and HFS being the most common ones [41]. This work suggested that in the Obs group, there were 2 cases of mild fatigue, 1 case of liver pain, 1 case of HFS, 2 cases of diarrhea, and 2 cases of fever, with an incidence of ARs of 16%. In the Ctrl group, there were 3 cases of mild fatigue, 2 cases of liver pain, 3 cases of HFS, 2 cases of diarrhea, and 4 cases of fever, with an incidence of ARs of 28%. The incidence of ARs in the Obs and Ctrl groups presented an obvious difference (*P* < 0.05). This indicates that the addition of sorafenib treatment on top of TACE can effectively reduce the occurrence of ARs in patients and demonstrates its safety and feasibility.

## Conclusion

100 patients with ALC admitted to the hospital from May 2020 to August 2022 were chosen and rolled into two groups using a random number table method: 50 patients in the Obs group and 50 patients in the Ctrl group. The patients in the Ctrl group received TACE treatment alone, while those in the Obs group received oral sorafenib treatment in addition to TACE. Meanwhile, it compared the basic information, DCR, QOL, immune indicators, and ARs of patients in different groups. The results disclosed that the addition of sorafenib treatment on top of TACE had significant short-term efficacy for ALC patients. It effectively improved the QOL of patients, enhanced their immune function, and reduced the incidence of ARs, demonstrating safety and feasibility. However, it was important to note that the number of ALC patients enrolled in this work was small, and they were all from the same hospital. Moreover, there was limited collection of prognostic data for the patients, which hindered the confirmation of the long-term efficacy of sorafenib treatment. Therefore, in future studies, it will select LC patient data from multiple hospitals and conduct a more in-depth analysis of the clinical application value of sorafenib. In summary, the findings of this work yielded reference for the design of ALC treatment regimens.

## Data Availability

All data generated or analysed during this study are included in this article. Further enquiries can be directed to the corresponding author.
